# The relative importance of resilience factors prior, during, and after stress-inducing exams in medical students

**DOI:** 10.1038/s41598-026-63690-x

**Published:** 2026-07-24

**Authors:** Pascal Schlechter, Paul O. Wilkinson, Philip Jefferies, Jessica Fritz

**Affiliations:** 1 Institute of Psychology, University of Münster, Münster, Germany; 2 Department of Psychiatry, University of Cambridge, Cambridge, UK; 3 Faculty of Science & Health, Atlantic Technological University, Castlebar, Ireland; 4 Institute of Psychology, University of Osnabrück, Osnabrück, Germany

**Keywords:** Resilience, Protective factors, Medical students, Natural stressor, Mental health, Health care, Psychology, Psychology, Risk factors

## Abstract

Resilience is a dynamic process of adapting to stress. Identifying modifiable resilience factors (RFs) remains challenging due to the difficulty of studying individuals before, during, and after a stressor in real time. We addressed this by investigating modifiable protective factors in the context of university exams, a predictable and impactful stressor, and focused on medical students to examine RFs in a vulnerable cohort under standardized conditions. In this exploratory study, we used data from the three-wave ‘RESIST’ Study involving 451 medical students from the UK. Our investigation covered three timepoints with differing levels of stress (1) a non-exam period, (2) an exam period, and (3) post-exams at the end of the term. We examined the distinct impact of 13 modifiable RFs at individual, family, and community levels. Specifically, we investigated their relative importance and their cumulative impact with regard to self-reported mental health symptoms at three timepoints. We found the highest relative importance rankings for self-esteem, brooding, and distress tolerance. While most individual resilience factors had negligible-to-small effects, the sum of all 13 effects was similar to the effects of self-reported mental health symptoms at the previous timepoint on themselves. Our findings shed light on the role of RFs, and their cumulative and timing effects, and their associations with exam-related self-reported mental health problems among medical students.

**Supplementary Information:**

The online version contains supplementary material available at 10.1038/s41598-026-63690-x.

## Introduction

Resilience is a dynamic process of adapting and bouncing back from adversity, trauma, or significant stressors, thereby reducing mental health risks^1–5^. A systematic review identified several specific potentially modifiable resilience enhancing factors for mitigating psychopathology risks following childhood adversity^6^. This review and general accounts of multisystemic resilience^7^ have emphasized the importance of considering a system of multiple resilience factors (RFs) operating on individual, family, and community levels to effectively reduce the risk of psychopathology after adversities.

Previous research in various populations has elucidated relevant relationships among RFs and their cumulative effects on mental health outcomes. For instance, network analysis and other system approaches have been employed to study resilience (e.g.,^8^). This body of literature suggests, for example, that childhood adversity may contribute to a dysfunctional system of RFs in 14-year-old adolescents^9^; or, that young people with mental health problems that do not interfere with good wellbeing (for example, due to being well-managed), may be better equipped with RFs than young people with no mental health problems but low wellbeing^10^. Moreover, a sense of coherence and family-level RFs seemed to be particularly important for mental health outcomes during the COVID-19 pandemic (an unexpected stressor) in the general German population^11^ (for a COVID-19 specific review, see^12^), and low brooding and high self-esteem emerged as the most relevant of 10 RFs predicting mental distress (without assessing specific stressors during that time) over the course of adolescence in a population cohort from the UK (Cambridgeshire)^13^.

Given the multitude of potential RFs, it is important to identify those that can be enhanced prior to, during, or in the aftermath of significant stress, and to examine whether their impact differs depending on these distinct time points in specific populations^14^. However, to date, studies systematically investigating the relative importance of specific RFs and their cumulative effects on mental health prior, during, and after a predictable and impactful stressor are a rarity. The deliberate induction of stress poses significant ethical and practical challenges for resilience research. In response, some studies have examined resilience during naturally occurring, unplanned stressors, such as earthquakes^15^ or the COVID-19 pandemic^11,12^. Yet, such designs are limited by their unpredictability, lack of standardization, and reduced control over timing and context^16^.

In the present study, we investigated exam stress in medical students, which represents a predictable, standardized, impactful, and ethically acceptable acute stressor. Therefore, this approach enables the prospective measurement of modifiable protective factors in the context of anticipatory academic stress and outcomes in a fairly controlled stress-context, at three defined time points: prior to, during, and after stress-inducing exams. While this is advantageous, it is important to clarify that exam stress differs from how resilience is typically conceptualized in the literature and that we specifically focus on modifiable protective factors in the context of anticipatory academic stress among medical students. Resilience studies often focus on acute, unexpected adversities, or chronic stressors^2,7,8,11,12,17,18^. Unlike acute, unexpected adversities (e.g., natural disasters, bereavement) or chronic stressors (e.g., prolonged parental drug use severe enough to cause significant family problems), university exams represent an anticipated stressor, often looming but bounded by a predetermined end date; students are aware of the stressor from the outset, with stress intensifying as exams approach and decreasing when exams have ended^19^ .

A meta-analysis examining 122,356 medical students across 43 countries reported a depressive symptom prevalence rate of 27.2%, which is higher than the one of their general population peers^20^. Additionally, elevated levels of anxiety and general distress were observed in medical students compared to population-representative samples^21^. Mental health problems increase the risk of medical students leaving courses^22^, which is especially important to address, given the costs of training doctors and the difficulties in retaining students and doctors in the medical profession^23^. Academic demands impose significant stress on medical students, peaking at the end of the semester due to upcoming exams, intensive preparation, and competition for grades^24^. Exams are highly relevant for academic careers and thus amplify stress levels^19,25,26^, as well as mental distress^19^. Therefore, a better understanding of factors that contribute to students’ resilience prior, during, and after exam stress is helpful.

Some studies have focused on specific modifiable protective factors among medical students such as social support at the University of North Dakota^27^ or coping at the University of Saskatchewan^28^. Other studies have examined resilience as trait-like factors in Australia, Canada, and the USA^29–31^ or discerned resilient personality profiles among medical students at the University of Queensland^32^. Moreover, a six-year longitudinal study has investigated how coping skills mitigated decreasing life satisfaction among medical students at four Norwegian universities^33^. This research has improved our understanding of protective factors among medical students. However, most studies have not assessed stress or adversity directly and no existing study has compared a set of established RFs from the literature^6^ to determine their relative importance among medical students in the specific context of exam stress. Since exam-related stress has a gradual onset with an acute exposure period (during exams), and a tapering off post-exams, this timeline may influence how RFs operate, possibly requiring different mechanisms of resilience before, during, and after the stressor. Table 1 details thirteen empirically supported RFs and their potential roles during exam stress. Additionally, there is a lack of investigation into the cumulative nature of several types of RFs during periods of stress versus no-stress. This is an important omission in the current literature, as such an approach enables the identification of specific pre-exam or during-exam RFs that could mitigate the effects of exam stress or promote recovery after exams.

**Table 1:** Importance of resilience enhancing factors in the context of exam stress.

Resilience factor	Importance in the context of exam stress
Individual-level resilience factors	
1. High Distress Tolerance	Enables students to withstand and manage heightened stress levels associated with exams^34^
2. Low Ruminative Reflection	Reduces overthinking and excessive self-reflection, allowing students to focus on task-specific aspects rather than dwelling on uncertainties^35^
3. Low Ruminative Brooding	Minimizes repetitive negative thinking, preventing the persistence of exam-related worries and promoting a more constructive approach to studying ^35^
4. High Self-Esteem	Enhances confidence and self-worth, providing a buffer against self-doubt and fostering a positive mindset during challenging exam periods^36^
5. High Cognitive Reappraisal	Encourages adaptive reinterpretation of stressors, helping students perceive exams as challenges rather than threats^5,37^
6. Low Expressive Suppression	Facilitates open expression of emotions, allowing students to seek support and cope effectively with the emotional strain associated with exams^5,37^
7. Low Aggression Potential	Mitigates interpersonal conflicts and hostility, creating a conducive environment for collaborative study and mutual support during exams^38^
Family-level resilience factors	
8. High Immediate Family Support	Offers a strong support system, providing emotional reassurance and practical assistance, contributing to a sense of security during exams ^39^
9. High Extended Family Support	Broadens the support network, offering additional resources and perspectives, which can be particularly beneficial during stressful exam periods^39^
10. High Family Cohesion	Fosters a sense of belonging, creating a stable environment that can positively influence a student’s emotional state during exams^40^
11. High Positive Parenting	Encourages a nurturing and encouraging parental style, promoting effective coping strategies in response to exam challenges^41^
12. High Parental Involvement	Involvement in a student’s academic journey provides a sense of guidance and mentorship, contributing to more support during exam stress^42^
Community-level resilience factors	
13. High Friendship Support	Cultivates a network of peers offering emotional and academic support, providing a crucial additional layer of resilience during the stress of exams^43^

This study addresses these gaps by conducting a broad examination of the relative and cumulative importance of RFs for medical students prior to, during, and in the aftermath of exam periods. We draw on data from the RESIST^19^, a cohort study that assessed medical students across three distinct periods: (1) a phase of moderate stress several months before exams within the university term (i.e., inducing some stress due to course requirements), (2) a phase of heightened stress during the exam period (i.e., due to exam preparation and due to taking the exams), and (3) a phase of low or moderate stress after exams (i.e., most students having holidays). We focused on medical students in one university with a large cohort to maximize consistency in exposure and reduce variability in stressor intensity. In this cohort, both perceived stress and self-reported mental distress have been observed to increase from the pre-exam period to the exam period and then decline after the exams to levels lower than pre-exams^19^. Our goal was to investigate how modifiable protective factors in the context of anticipatory academic stress may uniquely and cumulatively relate to self-reported mental health problems. Accordingly, we examined thirteen individual, family, and community RFs. To discern the unique contributions of each RF to self-reported mental health symptoms, we conducted a relative importance analysis of the included RFs. Given the specific context of exam stress that has not been previously examined in this manner, our research was exploratory.

## Methods

### Openness and transparency

The study protocol of this study has been published, which includes mean levels of sample self-reported mental health symptoms and perceived stress prior, during, and after exams^19^. This study protocol also includes some of the descriptive statistics we present here. The R Code for this study is available on the open science framework: https://osf.io/u7vfh/?view_only=189c1a8fbac246e89fbf7e59bc146e3f. Data and a codebook (excluding descriptive data) are available at: https://www.repository.cam.ac.uk/items/e7ac65b9-da6a-44a3-a233-0ade9296fba8.

### Participants and design

Data are drawn from RESIST^19^, a cohort study of 451 first- to sixth-year University of Cambridge medical students (57.4% females). In total, this student body comprised 1,464 individuals, that is, around 31% of the cohort took part in the study. The study included three timepoints: Timepoint 1 occurred during a non-exam period in the university term (February/March 2018), while timepoint 2 coincided with the end-of-year exam period (approximately April to June 2018, which was adapted to match the exam dates of the respective cohorts). At timepoint 2, students received questionnaires three weeks before their first final exam, and completion was required before attempting the final exam. This ensured uniform exposure to the stressor of exam preparation. Timepoint 3 took place after the exam period, at the end of the term for year 6 students (end of May to mid-July 2018, since they left the university afterwards) and during the summer vacation or autumn for year 1–5 students. At all timepoints, participants completed the survey with web-based questionnaires using REDCap^44^. Participants were at least 18 years old and received reimbursement in the form of web-based vouchers (£5 for timepoint 1, £7 for timepoint 2, and £5 for timepoint 3). Those who completed all three timepoints were entered into a prize draw to win one of five £50 vouchers. According to a power analysis not tailored to the present research question, the minimum sample size target was 225 participants^19^. This calculation was based on a Gaussian regression-based model including nine risk factors and one outcome variable, following recommendations of at least five observations per estimated parameter. Participants provided informed consent and the study was approved by the Cambridge University Research Ethics Committee (PRE.2017.096). The study was conducted in accordance with relevant guidelines as well as the Declaration of Helsinki.

### Outcome variables

**Mental health.** Self-reported general mental health was assessed with the 12-item version of the General Health Questionnaire (GHQ-12)^45^. Items tap into, for example, concentration problems, sleep, and happiness, and are measured on a 4-point scale, ranging from 0 (“*not at all*”) to 3 (“*completely*”). Items ask, for example: “*Have you recently been able to concentrate on what you’re doing?”.* Overall, the GHQ-12 shows good psychometric properties (for reviews, see^46,47^). A cut-off above 14 indicates probable levels of mental health problems^45^.

### Resilience factors

A detailed conceptual overview of the content of the RFs can be found in Table 1.

**Distress tolerance**. Distress tolerance was evaluated with the subjective appraisal of distress 6-item subscale from the Distress Tolerance Scale^48^. Items encompass aspects such as the acceptability of being upset. Items were rated on a 5-point Likert scale from 1 (“*strongly agree*”) to 5 (“*strongly disagree*”). The scale asks to “*think of times that you feel distressed or upset*”. An example item reads: “*Feeling distressed or upset is unbearable to me*”. Prior research demonstrated good reliability of the Distress Tolerance Scale^48^.

**Ruminative reflection.** Ruminative reflection was measured with the 5-item reflective rumination subscale from the Ruminative Response Scale (RRS)^49^. Items encompass attempts to comprehend the reasons behind experiencing negative mood. Items were rated on a 4-point Likert scale ranging from 1 (“*never*”) to 4 (“*always*”). The scale asks “*please indicate what you generally do*”. An example item reads: “*analyse recent events to try to understand why you are depressed*”. Psychometric properties of the RSS have been confirmed in an undergraduate sample^50^.

**Ruminative brooding.** Ruminative brooding was also assessed with the RRS using the 5-item brooding subscale with the same response format as for ruminative reflection^49^. This subscale taps into brooding tendencies, such as contemplating that situations do not improve. An example item reads: *“think “What am I doing to deserve this?*”.

**Self-esteem.** The Rosenberg Self-Esteem Scale (RSES)^51^ assesses general self-esteem with ten items. The RSES asks for instance whether people are satisfied with themselves, by using a four-point Likert scale ranging from 0 (“*strongly disagree*”) to 3 (“*strongly agree*”). The RSES asks for self-esteem in general. An example item reads: “*On the whole, I am satisfied with myself*.”. A one-factor model is psychometrically supported (for a meta-analysis, see^52^).

**Cognitive reappraisal.** Cognitive reappraisal was assessed with the Emotion Regulation Questionnaire (ERQ)^53^, using the six item cognitive reappraisal subscale of the 10-item self-report questionnaire. The six items assess habitual use of cognitive reappraisal on a 7-point Likert scale ranging from 1 (“*strongly disagree*”) to 7 (“*strongly agree*”). Items ask for general patterns of reappraisal. An example item reads:” *When I want to feel more positive emotion (such as joy or amusement), I change what I’m thinking about*”. Psychometric properties of the ERQ were good across different samples in prior research^54^.

**Expressive suppression.** The ERQ^53^ was also used to assess expressive suppression, as the remaining four items tap into the habitual use of expressive suppression as emotion regulation strategy (using the same Likert scale as for cognitive reappraisal). Items assess general patterns of expressive suppression. An example item reads: “*I keep my emotions to myself*”.

**Low aggression potential.** The 12-item Brief Aggression Questionnaire (BAQ)^55^ assesses aggression levels including physical aggression, verbal aggression, anger and hostility. The BAQ uses a 5-point response scale ranging from 1 (“*extremely uncharacteristic of me*”) to 5 (“*extremely characteristic of me*”). Items ask for general patterns of aggression. An example item reads: “*Given enough provocation, I may hit another person.*” Reliability and validity of the BAQ have been demonstrated in prior work^56^.

**Immediate family support.** We used the 6-item version of the Perceived Social Support from Family Scale (PSS-FA)^57^. The PSS-FA taps into emotional support and support with problem solving provided by family members with a dichotomous (yes = 1/no = 0) scale. Items assess general patterns of family support. An example item reads: “*Members of my family seek me out for companionship*”. Reliability and validity of the PSS-FA have been demonstrated in previous research^58^.

**Extended family support.** To assess extended family support, we applied the 13-item Kinship Social Support Measure^59^, which assesses support such as advice from relatives during decision-making or when having problems. Items were rated on a 4-point scale, ranging from 1 (“*strongly disagree*”) to 4 (“*strongly agree*”). Items ask for general patterns of family support. An example item reads: "*When I’m worried about something I look to my relatives for advice*.”. The Kinship Social Support Measure has good internal consistencies and validity^59^.

**Family cohesion.** We assessed family cohesion with the 5-item family cohesion subscale of the Self-Report Family Inventory Version II^60^. The scale taps into family cohesion, for instance, by asking whether individuals prioritize time spent with family over socializing with others on a scale from 1 (“*fits our family very well*”) to 5 (“*does not fit our family*”). Items ask for general patterns of family cohesion. An example item reads: “*Family members pay attention to each other’s feelings*”. Reliability and validity have been shown across different groups^61^.

**Positive parenting.** We used the 6-item positive parenting subscale of the Alabama Parenting Questionnaire (APQ)^62^, which probes aspects like parental encouragement (e.g., compliments or praise from parents). Response options ranged from 1 (“*never*”) to 6 (“*always*”). Students were asked to reflect on a time when they were living together with their parents when responding to this measure. An example item reads: “*You have a friendly talk with your mom*”. Factorial validity and reliability have been established for the APQ^63^.

**Parental involvement.** To assess parental involvement, we used the 10-item parental involvement subscale of the APQ^62^. This subscale taps into family activities and parental school involvement and uses the same Likert-scale as for positive parenting. Again, students were asked to reflect on a time when they were living together with their parents. An example item reads: “*Your mom asks you what your plans are for the coming day*”.

**Friendship support.** We measured friendship support with the 6-item abbreviated version of the Perceived Social Support from Friends Scale (PSS-FR)^57^. The PSS-FR taps into emotional support (e.g., moral support and having companionship) provided by friends using a dichotomous (yes/no) scale, leading to a maximum score of 6. Items ask for general patterns of friendship support. An example item reads: “*My friends give me the moral support I need*.”. Reliability and validity of the PSS-FA have been demonstrated in previous research^58^.

### Missingness

The highest level of missing data was 39% based on data present for 273 (of the 451) participants for timepoint 2. Gender (male gender), ethnicity (non-white), medication use, and higher global stress contributed to missingness (see^19^ for a detailed analysis of predictors of missingness). Therefore, we treated data as missing at random^64^. We adopted a multiple imputation strategy using the *mice* package in R. To this end, we imputed a total of 40 datasets (based on 39% missingness) and pooled the results. During the imputation process, we incorporated all demographic characteristics specified in Table 2 as auxiliary variables to enhance the accuracy of our imputed values. We conducted sensitivity analyses based on full cases (see Supplemental Table s1).

**Table 2:** Demographic characteristics of all 451 participants.

Characteristics	n (%)
Academic year	
First year	93 (20.6)
Second year	83 (18.4)
Third year	66 (14.6)
Fourth year	88 (19.5)
Fifth year	56 (12.4)
Sixth year	65 (14.4)
Gender	
Female	259 (57.4)
Male	185 (41.0)
Prefer not to say	6 (1.3)
Missing	1 (0.2)
Age (years)	
18–20	170 (37.7)
21–23	196 (43.5)
24–26	65 (14.4)
≥ 27	17 (3.8)
Missing	3 (0.7)
Ethnicity	
White	263 (58.3)
Non-White	184 (40.8)
Missing	4 (0.9)
Therapeutic treatment (in the 6 months before occasion 1)	
No	390 (86.5)
Yes	61 (13.5)
Psychopharmaceutic treatment (in the 6 months before occasion 1)	
No	402 (89.1)
Yes	49 (10.9)
Education (higher education after secondary school)	
Mother	
Yes	359 (79.6)
No	88 (19.5)
Unknown	4 (0.9)
Father	
Yes	369 (81.8)
No	77 (17.1)
Unknown	4 (0.9)
Missing	1 (0.2)

This table is taken from Fritz et al.^19^. Some numbers do not add up to 100% due to missing values.

### Analysis strategy

Analyses were performed in R version 4.3.0^65^. Given the high number of statistical tests, we placed 99.9% confidence intervals around the estimates, corresponding to α = 0.001. We calculated the *relative importance* of our explanatory variables (13 RFs) using three model combinations (i.e., RFs explaining mental health for all three time point combinations: T1→T2; T1→T3; T2→T3). Relative importance analysis is well-suited for the present study because it addresses potential concerns regarding multicollinearity among predictors by partitioning explained variance in a way that is robust to intercorrelations between explanatory variables^66^. All resilience factors were simultaneously included in each model, ensuring that relative importance estimates reflect their unique contributions to explained variance (for details on differences and similarities with dominance and relative weight analysis, see^66^). Of note, we calculated these models in three different ways, resulting in three slightly differing sets of results:

***RI A Models: Models with only RFs as predictors.*** In this first set of three models, we only examined the effect of RFs on self-reported mental health symptoms without considering autoregressive effects of self-reported mental health symptoms. Thus, the *R*^2^ is distributed only among the RFs. Therefore, those analyses examine how well RFs can explain *absolute levels* in self-reported mental health symptoms on the respective other time point. More specifically, those analyses address questions such as “*What before-exam RF is most strongly associated with mental health levels during exams?*”.

***RI B Models: Models with autoregressive effects of self-reported mental health symptoms and RFs as predictors.*** This second set of models includes the autoregressive effect of mental health as an additional predictor (i.e., self-reported mental health symptoms at a previous time point explaining themselves at a later time point). Thus, the *R*^2^ is distributed among the autoregressive effect and the RFs. This helps compare the explanatory effect sizes of self-reported mental health symptoms and RFs. Accordingly, these analyses can answer research questions such as “*How strongly are the different before-exams RFs associated with self-reported mental health symptoms during exams, compared to the degree to which self-reported mental health symptoms before-exams explains self-reported mental health symptoms during exams (i.e., the autoregressive effect)?*”.

***RI C Models: Models, conditioned on autoregressive effects of self-reported mental health symptoms, with RFs as predictors.*** In this third set of models, the autoregressive effect of self-reported mental health symptoms was conditioned upon in the models because autoregressive effects usually explain most variance in outcome variables. That is, the autoregressive factor was always included as first variable in the models. The remaining *R*^2^, which is not explained by the autoregressive effect, is then distributed among the RFs. Therefore, these analyses examine the *relative contributions* of different RFs to the *change in self-reported mental health symptoms* on the respective other time point. More specifically, they can answer research questions such as “*What before-exam RF is most strongly associated with changes in self-reported mental health symptoms from before to during exams?*”.

This led to a total of 9 models. To calculate these 9 models, we used the LMG method^67^, which calculates the relative contribution of each explanatory variable to the *R*^2^, taking into account all possible orders in which explanatory variables can appear in the model. More specifically, to determine the individual proportion of *R*^2^, the lmg approach addresses the issue of effect-size-dependency given variable order, by averaging the unique effects of explanatory variables over all possible orderings, and thereby identifies the (relative) importance ranking of the explanatory variables (for explaining the outcome variable). We used the *relaimpo* package in R to conduct our analyses^68^. Following typical conventions^69^, we interpreted an effect size rounded to *R*^2^ < 0.015 as negligible, 0.015 ≤ *R*^2^ < 0.09 as small, 0.09 ≤ *R*^2^ < 0.25 as moderate, and *R*^2^ ≥ 0.25 as large. To increase certainty around the relative importance estimates and to compare the RFs against each other, we constructed 99.9% confidence intervals via bootstrapping using 1000 iterations.

## Results

### Descriptive statistics

In total, 451 participants participated at timepoint 1, with dropouts in subsequent timepoints (timepoint 2: *n* = 275; timepoint 3: *n* = 283). Of the 451 participants at timepoint 1, 71.8% participated at least twice. For timepoint 2, 61.0% (275 participants) were involved, and 14.9% (41 individuals) did not continue to timepoint 3. On timepoint 3, 62.8% (283 individuals) took part, of whom 17.3% (49 individuals) had not participated in timepoint 2. Notably, 51.9% (234 individuals) were involved in all three timepoints. Demographic characteristics of all participants can be found in Table 2. Table 3 details descriptive statistics of all variables, including mean levels of self-reported mental health symptoms, internal consistency and retest-reliability/stability across the different timepoints. At timepoint 1, 37.98% of participants scored above the GHQ-12 cut-off of 14. At timepoint 2, 49.45% exceeded the cut-off, and at timepoint 3, 25.18% scored above the threshold. This is consistent with the mean level trajectories of the GHQ-12. At all time-points, internal consistencies were acceptable to good for all scales (0.73–0.94). For the RFs, retest-reliability ranged from (mostly) acceptable to good (0.51–0.87). Correlations among the RFs at a given timepoint can be found in Supplemental Figs. s1–s3.

**Table 3:** Descriptive statistics and retest-reliabilities for all three waves T1–T3.

	T1				T2				T3				Retest-reliability/stability		
	*M*	*SD*	α	${\upomega}_{t}$	*M*	*SD*	α	${\upomega}_{t}$	*M*	*SD*	α	${\upomega}_{t}$	r_t1t2_	r_t1t3_	r_t2t3_
Mental Health	25.40	5.82	.88	.89	27.38	6.09	.89	.89	23.31	5.93	.90	.91	.53 [.38: .66]	.30 [.11; .47]	.22 [.00; .41]
High distress tolerance	20.41	5.41	.82	.82	20.05	5.47	.84	.85	20.01	5.49	.83	.83	.78 [.69; .85]	.70 [.58; .79]	.71 [.59; .80]
Low ruminative reflection	13.81	3.34	.75	.76	13.66	3.22	.76	.76	13.69	3.43	.79	.80	.67 [.54; .77]	.58 [.44; .70]	.68 [.54; .78]
Low ruminative brooding	14.22	3.25	.75	.76	14.10	3.29	.79	.80	14.27	3.30	.77	.78	.67 [.54; .77]	.63 [.49; .73]	.68 [.54; .78]
High self-esteem	47.34	12.13	.93	.93	45.87	12.4	.94	.94	47.54	11.25	.92	.92	.87 [.81; .91]	.82 [.74; .87]	.78 [.68; .86]
High cognitive reappraisal	28.35	6.10	.83	.83	28.38	6.04	.87	.87	28.29	6.53	.88	.88	.62 [.48; .73]	.51 [.34; .64]	.64 [.49; .75]
Low expressive suppression	16.91	4.98	.75	.78	17.12	4.82	.73	.76	16.98	4.75	.76	.78	.75 [.65; .83]	.64 [.51; .74]	.76 [.65; .84]
Low aggression potential	76.67	13.38	.78	.79	74.96	13.58	.80	.81	74.82	13.37	.79	.78	.85 [.78; .90]	.79 [.70; .85]	.83 [.74; .88]
Immediate family support	4.35	2.07	.88	.88	4.53	1.87	.83	.83	4.51	1.93	.85	.85	.80 [.72; .86]	.79 [.70; .85]	.80 [.71; .87]
Extended family support	35.46	8.05	.92	.92	35.29	7.48	.91	.91	35.35	7.78	.93	.93	.81 [.73; .87]	.82 [.75; .88]	.86 [.79; .91]
High family cohesion	16.85	4.84	.86	.87	16.48	4.46	.84	.85	16.46	4.69	.87	.88	.85 [.78; .89]	.80 [.71; .96]	.84 [.76; .89]
High positive parenting	21.78	5.16	.87	.87	21.96	5.11	.88	.88	21.46	4.96	.88	.88	.82 [.75; .88]	.73 [.63; .81]	.83 [.76; .89]
High parental involvement	38.00	7.48	.87	.87	38.53	7.46	.89	.90	38.02	7.11	.87	.87	.82 [.75; .88]	.79 [.71; .86]	.83 [.75; .89]
High friendship support	4.58	1.76	.80	.80	4.59	1.79	.81	.81	4.79	1.65	.79	.79	.77 [.68; .84]	.61 [.47; .72]	.73 [.61; .81]

M = Mean; SD = Standard deviation, α = Cronbach’s α, ${\upomega}_{\mathrm{t}}$ = Omega Total.

### Relative importance analyses

***RI A Models (RF-only models).*** These findings are based on our models that only include the effect of RFs on self-reported mental health symptoms (Table 4), indicating how well RFs can explain *absolute levels* in self-reported mental health symptoms. Self-esteem had the highest or second highest contribution in all models, with small sized effects (ranging from 2 to 8%). Brooding and distress tolerance also ranked among the highest contributors, with negligible-to-small effects (ranging from 1 to 5%). High immediate family support emerged as the most important variable (4%) in the T1→T3 model, and positive parenting had a small effect in the T1→T2 model (2%). Apart from this, all other RFs contributed only negligible variance to the models (0 or 1%). The total explained variance for self-reported mental health symptoms ranged from *R*^2^ = 0.06–0.24.

**Table 4:** Relative Importance of the resilience factors on self-reported mental health symptoms outcome.

Model	RI A Models (RF-only models)			RI B Models (RF + autoregressive models )			RI C Models (RF effects on change controlling for prior mental health)		
Timepoints Explained total variance	T1—> T2 R^2^ = .24	T1—> T3 R^2^ = .11	T2—> T3 R^2^ = .06	T1—> T2 R^2^ = .29	T1—> T3 R^2^ = .13	T2—> T3 R^2^ = .07	T1—> T2 R^2^ = .28	T1—> T3 R^2^ = .10	T2—> T3 R^2^ = .07
Sum of all resilience factors	–	–	–	.18	.10	.06	.07	.06	.04
Baseline mental health	–	–	–	.11 [.06; .26]	.03 [.00; .13]	.01 [.00; .11]	.21[.15; .28]	.04 [.01; .08]	.03 [.01; .10]
High distress tolerance	.05 [.01; .13]	.01 [.00; .10]	.01 [.00; .09]	.03 [.01; .10]	.01 [.00; .08]	.01 [.00; .08]	.01 [.00; .04]	.00 [.00; .06]	.01 [.00; .07]
Low ruminative reflection	.01 [.00; .05]	.01 [.00; .09]	.00 [.00; .06]	.01 [.00; .04]	.01 [.00; .08]	.00 [.00; .06]	.00 [.00; .04]	.01 [.00; .07]	.00 [.00; .07]
Low ruminative brooding	.05 [.01; .15]	.01 [.00; .05]	.01 [.00; .05]	.03 [.01; .12]	.01 [.00; .04]	.01 [.00; .05]	.01 [.00; .07]	.00 [.00; .04]	.01 [.00; .05]
High self-esteem	.08 [.03; .17]	.02 [.00; .13]	.02 [.00; .11]	.06 [.02; .14]	.02 [.00; .11]	.01 [.00; .09]	.01 [.00; .07]	.00 [.00; .07]	.01 [.00; .06]
High cognitive reappraisal	.01 [.00; .06]	.00 [.00; .04]	.00 [.00; .06]	.01 [.00; .05]	.00 [.00; .04]	.00 [.00; .06]	.00 [.00; .03]	.00 [.00; .06]	.00 [.00; .06]
Low expressive suppression	.00 [.00; .05]	.00 [.00; .06]	.01 [.00; .08]	.00 [.00; .04]	.00 [.00; .06]	.01 [.00; .08]	.00 [.00; .04]	.00 [.00; .05]	.01 [.00; .07]
Low aggression potential	.00 [.00; .04]	.00 [.00; .05]	.00 [.00; .07]	.00 [.00; .04]	.00 [.00; .05]	.00 [.00; .07]	.00 [.00; .04]	.00 [.00; .05]	.00 [.00; .06]
High immediate family support	.01 [.00; .07]	.04 [.00; .11]	.00 [.00; .06]	.01 [.00; .06]	.03 [.00; .11]	.00 [.00; .06]	.00 [.00; .05]	.02 [.00; .09]	.00 [.00; .06]
High extended family support	.00 [.00; .03]	.00 [.00; .04]	.00 [.00; .04]	.00 [.00; .03]	.00 [.00; .04]	.00 [.00; .04]	.00 [.00; .03]	.00 [.00; .04]	.00 [.00; .05]
High family cohesion	.00 [.00; .04]	.01 [.00; .07]	.00 [.00; .09]	.00 [.00; .03]	.01 [.00; .07]	.00 [.00; .09]	.00 [.00; .03]	.01 [.00; .07]	.00 [.00; .09]
High positive parenting	.02 [.00; .06]	.01 [.00; .06]	.00 [.00; .05]	.02 [.00; .06]	.01 [.00; .05]	.00 [.00; .05]	.01 [.00; .05]	.01 [.00; .06]	.00 [.00; .05]
High parental involvement	.01 [.00; .08]	.00 [.00; .05]	.00 [.00; .06]	.01 [.00; .07]	.00 [.00; .05]	.00 [.00; .06]	.00 [.00; .05]	.00 [.00; .05]	.00 [.00; .06]
High friendship support	.00 [.00; .04]	.00 [.00; .05]	.01 [.00; .04]	.00 [.00; .03]	.00 [.00; .04]	.01 [.00; .04]	.00 [.00; .02]	.00 [.00; .03]	.00 [.00; .05]

Number in brackets refer to bootstrapped 99.9% confidence intervals based in 1,000 iterations. Relative Importance of the resilience factors without autoregressive factors in regression models (RI A Models); Relative Importance of the resilience factors and autoregressive effect of baseline mental health in regression models (RI B Models); Relative Importance of the resilience factors with autoregressive effect of baseline mental health always accounted for (RI C Models).

Effect size rounded to R^2^ < 0.015 are considered as negligible, 0.015 ≤ R^2^ < 0.09 as small, 0.09 ≤ R^2^ < 0.25 as moderate, and R^2^ > 0.25 as large.

***RI B Models (RF* + *autoregressive models):*** These findings are based on our models that include the autoregressive effect of self-reported mental health symptoms and RFs as predictors (Table 4), which help compare the explanatory effect sizes of self-reported mental health symptoms and RFs. The autoregressive effect held the highest or joint highest relative importance in all models. In the T1→T2 model, mental health had a moderate predictive effect; three individual level RFs (self-esteem, distress tolerance, low brooding) and one family-level RF (positive parenting) had small effects. All other RFs had negligible effects. In the T1→T3 model, mental health had only a small predictive effect; one individual level RFs (self-esteem) and one family-level RF (immediate family support) had small effects. All other RFs had negligible effects only. In the T2→T3 model, mental health and all RFs had negligible effects only. Note that confidence intervals overlapped, so relative importance ranks are only descriptive trends. Importantly, in all three models, the sum of the explained variance of the RFs—or put differently, the cumulative effect of the 13 RFs—was larger than the amount of explained variance of the autoregressive effect of self-reported mental health symptoms. The total explained variance for self-reported mental health symptoms ranged from *R*^2^ = 0.07–0.29.

***RI C Models (RF effects on change controlling for prior self-reported mental health symptoms).*** These findings are based on our models that include the autoregressive effect of self-reported mental health symptoms being conditioned upon and RFs as predictors (Table 4), which help compare how well individual RFs can explain *change* in self-reported mental health symptoms. All except one of the effect sizes of RFs were negligible. Self-esteem, distress tolerance, and brooding ranked among the most important contributors in the T1→T2 and T2→T3 models, but also only contributed negligible variance to the models. High immediate family support had the highest relative importance in the T1→T3 model, obtaining a small effect, and thereby the largest effect of all RF in this set of models. Note that confidence intervals overlapped again, so relative importance ranks are only descriptive trends. The total explained variance for self-reported mental health symptoms ranged from *R*^2^ = 0.07–0.28.

### Sensitivity analysis

A sensitivity analysis based on complete cases is presented in Supplemental Table s1. The overall pattern of results was consistent with those obtained from the analyses using imputed data. However, some effect sizes, particularly the autoregressive effects, were slightly larger in the complete-case analysis compared to the imputed-data analysis.

## Discussion

To capture the dynamic nature of resilience, it is critical to measure RFs and outcomes across multiple time points: ideally before, during, and after exposure to a stressor. To examine a predictable and impactful acute stressor in a standardized way, we examined modifiable protective factors in medical students in a moderately stressful period before exams, a heightened stress period during exams, and a low-to-moderate stress period post-exams. In an exploratory study, we examined the relative and cumulative impact of RFs on self-reported mental health symptoms at subsequent timepoints.

Of all 13 RFs, self-esteem, brooding, and distress-tolerance had the highest relative importance for self-reported mental health symptoms, particularly from pre-exams to the exam period. However, most confidence intervals overlapped, so these are descriptive trends. Of note, relative importance patterns for RFs were similar for both explaining change in and absolute levels of self-reported mental health symptoms. However, in the RI C models (RF effects on change controlling for prior mental health) RFs contributed negligible additional explanatory information once prior symptom levels were fully accounted for. Our findings further suggest that pre-exam RFs explained more variance in self-reported mental health symptoms during exams, compared to pre-exam RFs or RFs during exams explaining post-exam outcome levels. While the effects of individual RFs were almost always negligible or small, the sum of the explained variance of the individual RFs was larger than the explained variance of the autoregressive effect of self-reported mental health symptoms in all but one model (RI C model for T1 → T2); and most of the models that only contained RFs as predictors had a moderate total variance explained by the combination of RFs. This pattern suggests that RFs, when considered together, account for a relevant proportion of variance in subsequent self-reported mental health symptoms. The comparison with autoregressive effects should be interpreted with caution. Cumulative effects reflect the aggregated contribution of multiple, partly correlated predictors rather than a direct or formal test of the relative importance of a “resilience system” versus the stability of self-reported mental health symptoms. Accordingly, the findings indicate that a combination of RFs provides additional explanatory information beyond individual factors, alongside autoregressive effects.

Our finding that self-esteem, distress tolerance, and brooding consistently contributed to self-reported mental health symptoms beyond autoregressive effects in some RI B models aligns with several prior studies conceptualizing self-esteem, distress tolerance, and brooding as transdiagnostic factors^70–74^. Although the effect sizes of these three RFs were predominantly negligible-to-small and confidence intervals were overlapping with other resilience factors, they were significantly related to self-reported mental health symptoms and change therein in both our study and another student study^75^. However, in the RI C models (RF effects on change controlling for prior mental health), effects were negligible across all RFs and time intervals.

The high ranking of self-esteem across models suggests that students with a positive self-perception may be better equipped to navigate challenges associated with exams^36,74^. Higher self-esteem may act as a buffer, potentially helping individuals maintain a more stable belief in their capabilities and a more constructive approach to managing stress during exams. Moreover, individuals with a greater tolerance of distressing situations have been found to experience lower levels of self-reported mental health symptoms during exams^34,71^. The role of brooding rumination underscores the potentially negative impact of dwelling on negative thoughts and feelings in the context of exams^70,72^.

High immediate family support and positive parenting, both factors on the family-level, were ranked high in some models. Yet overall, the individual-level factors self-esteem, distress tolerance, and brooding rumination displayed slightly larger effects than family- or community-level RFs. However, most confidence intervals were overlapping. The lower ranking of family- or community-level RFs may be attributable to various factors specific to the academic environment. Family members may be physically distant, especially if students are attending a university away from their homes. Physical separation might limit the immediate availability of family-based support during intense exam periods; or alternatively, if family members have not had the opportunity to attend university themselves, they may struggle to fully understand the different aspects of exam stress at university. In addition, family-level indicators were assessed retrospectively, reflecting earlier periods when students lived at home, which may limit their temporal alignment with current exam-related stress. Friendships formed at the university might not always translate into robust support systems, particularly during exam stress, as friends could be facing similar academic pressures and may not be readily available for support. Moreover, sometimes friends have to directly compete for grades, and thus academic challenges can be highly personal^76^. However, in responding to these items, medical students may not necessarily refer to friends at the university as opposed to friends elsewhere. Thus, while social support may remain important, the nature of exam-related stress is centered around individual academic performance, expectations, and self-evaluation, which may to some degree explain these findings. Moreover, while certain RFs such as self-esteem may have broader, cross-contextual effects, other factors like social support may be highly specific to particular forms of stress or adversity, underscoring the importance of further examining generalizable and context-dependent mechanisms in resilience processes.

Our findings additionally suggest that RFs prior to the high-stress period appear to explain more variance in how well individuals cope with the challenges of exam stress and (related) self-reported mental health symptoms, than on recovery after exams. This underscores the relevance of establishing or strengthening protective factors during term time, before students face intense academic pressure. This also suggests that addressing protective factors solely during the examination period, or post-exams for recovery purposes, may have a less significant impact on mitigating self-reported mental health symptoms—yet, this conjecture needs direct testing in future research. Of note, during post-exam periods, a natural improvement in self-reported mental health symptoms is expected once the stressor has passed, which likely contributes to the smaller effect sizes. In this context, smaller effect sizes are meaningful, as they reflect the diminishing influence of modifiable factors when external stressors are removed. Thus, including distinct time points before, during, and after exam stress seems to capture how psychological responses and RFs change across anticipation, peak stress, and recovery. This temporal structure may also apply to other forms of scheduled or anticipatory stress such as job interviews, planned surgeries, or divorce proceedings that follow a similar trajectory of build-up, acute challenge, and resolution.

While, in line with prior research^77^, many RFs had on their own negligible effects, they together resulted in small-to-moderate total effects. Thus, some individual RFs may not be particularly predictive of subsequent mental health outcomes. At the same time, the effects of RFs may differ among individuals. For instance, some people may benefit more from self-esteem and less from friendship support, while for others, the opposite may be true. As a result, these differences may average out when looking at the overall effects^77^. Importantly, the sum of the explained variance of the RFs explained a similar or slightly higher level of variance than the autoregressive effects of self-reported mental health symptoms, in all but one model. This is relevant because individual RFs typically exhibit small effect sizes that do not exceed the influence of autoregressive self-reported mental health symptoms effects^2^. Therefore, our study suggests that the combined impact of an overarching complex system of RFs is potentially meaningful when examining groups of people, highlighting the importance of considering multiple RFs rather than focusing on single ones. In contrast, when examining individual people, it may be more beneficial to focus on those RFs that are most relevant to the specific person.

In the RI B models (RF + autoregressive models), RFs were evaluated alongside the autoregressive effect of self-reported mental health symptoms, allowing them to share explanatory variance. These models therefore examine the extent to which RFs are associated with subsequent levels of self-reported mental health symptoms while accounting for prior symptom levels. In contrast, in the RI C models (RF effects on change controlling for prior mental health), the autoregressive effect was conditioned upon first, leaving RFs to account only for the residual change in self-reported mental health symptoms that could not be explained by self-reported mental health symptoms themselves. Because autoregressive effects typically explain a substantial proportion of variance in longitudinal mental health outcomes, the RI C models represent a considerably more stringent test of RF effects. Due to this difference in variance partitioning, the effect sizes for RFs were higher in the RI B models (RF + autoregressive models) than in the RI C models (RF effects on change controlling for prior mental health). Put differently, RFs contributed only negligible additional variance in the RI C models (RF effects on change controlling for prior mental health), whereas their effects appeared larger in the RI B models (RF + autoregressive models), although effects remained negligible-to-small for single RFs. This indicates that RFs (together) may be able to explain self-reported mental health symptom levels at subsequent time points, but their unique ability to predict *residual changes* in self-reported mental health symptoms seems limited.

### Recommendations for future research

Our study may inform future research. Future studies should refine how stress exposure is conceptualized and measured. Although exam periods provide a structured stressor, variability in perceived workload, difficulty, and concurrent life stress may still influence outcomes and should be explicitly assessed in future studies. Second, multi-method approaches to RFs are needed. Incorporating behavioral, physiological, or ecological momentary measures would reduce reliance on self-report data and provide a more robust assessment of resilience processes. Third, future studies should use intensive longitudinal or experience sampling designs to capture within-person dynamics. This would allow examination of whether RFs operate as stable between-person differences or fluctuate meaningfully over short periods of stress exposure^78^. Fourth, stronger causal designs are needed to clarify temporal ordering, for example through experimental approaches, to test whether changes in RFs precede changes in self-reported mental health symptoms and whether intervention timing matters. Finally, replication across different academic and non-academic contexts is necessary to determine whether the observed pattern of RF importance is specific to medical students under exam stress or generalizes to other forms of stress exposure.

### Strengths and limitations

This study exhibits several strengths including the opportunity to systematically examine mental health in a medium-sized sample across three relevant time points: pre-exam, during exam, and post-exam periods. Therefore, this prospective natural study design enables a nuanced understanding of the impact of an anticipated and commonly occurring stressor, namely end-of-the-year exams among medical students.

However, the study also has several limitations. Findings from our secondary data analysis may not generalize to other forms of stress, particularly acute or chronic stressors that differ from the predictable and anticipatory exam context. In our study, we only examined exam stress among medical students but not other types of exam stress in other student or trainee populations. Also, acute, unexpected events or ongoing chronic stress may relate to different resilience processes. In addition, it remains unclear whether our results generalize to contexts with a greater severity of stress. Given the exploratory nature of our secondary analyses, the findings should be interpreted with caution and require replication in independent samples to assess their robustness. However, our study examined empirically derived RFs that have been identified across diverse populations^6^. Thus, while stressors faced by medical students—such as exam-related pressures—may potentially be unique, the underlying mechanisms of resilience may perhaps extend beyond this group. Future research should explore the generalizability of these findings across different student populations and high-stress professions to elucidate this. In this regard, we do not have access to information on whether non-participants differed systematically from participants on any demographic variables. However, available admissions statistics for Cambridge medicine courses (https://www.undergraduate.study.cam.ac.uk/apply/before/application-statistics) suggest that women may have been somewhat overrepresented in the present sample relative to the underlying student population. Specifically, females comprise usually around 50% of the medical student intake, compared to 57.4% in the present sample. At the time these cohorts were admitted to Cambridge, ethnicity data at the course level were not systematically analyzed and published.

While we have addressed missing data, unmeasured variables in the current study may have also contributed to attrition, and, since they were not assessed, they could not be addressed through imputation. Yet, sensitivity analyses indicated that results were robust across imputed and complete-case data, enhancing confidence in our overall conclusions. Furthermore, causality, other than temporal precedence, cannot be established in our observational study. While RESIST assesses self-reported mental health symptoms across distinct time points, its temporal frame lacks granularity. Using Ecological Momentary Assessment studies in future research could offer a more detailed understanding of how RFs operate in the day-to-day experiences of students during exam periods^79,80^. Moreover, there is some uncertainty regarding the timing of the post-exam assessment to capture full recovery from exam-related stress and self-reported mental health symptoms. While the post-exam assessment was set after the expected resolution of acute stress, there is no empirical evidence on how long exam stress typically impacts self-reported mental health symptoms. Moreover, the post-exam assessment was not uniformly timed across participants. Although it was the period following the high-stress exam phase for all students, the actual timing of this assessment, and the length of post-exam vacation, varied by academic year and scheduling constraints. Consequently, the post-exam assessment likely captures a heterogeneous set of contextual conditions rather than a clearly defined and comparable post-stress recovery period. In addition, it would also have been valuable to include environmental RFs, such as living conditions or social environments, to provide a more comprehensive understanding of influences on resilience. A further limitation is that several predictor measures exhibit partial conceptual overlap with mental health outcomes. For example, items from the ruminative brooding subscale (e.g., “*I think about why I can’t handle this better*”) and the Brief Aggression Questionnaire (e.g., anger/hostility) measure some general aspects of mental health, which may have inflated the observed associations. Yet, because GHQ was controlled for in most models, a substantial proportion of variance associated with generalized distress was already partialled out, thereby reducing concerns regarding construct redundancy. Further, the relatively modest amount of explained variance observed across models, and the greater stability of RFs than self-reported mental health, suggest that this overlap is at least limited and unlikely to fully account for the findings, yet future work should prioritize measures with stronger discriminant validity.

## Conclusion

Our study points to the relative importance of RFs in the context of exam stress. The findings shed light on the role of pre-exam RFs including self-esteem, distress tolerance, and brooding and their associations with exam-related self-reported mental health symptoms as well as change therein. Although individual-level factors explained slightly more variance than family- and community-level factors, on the group-level, the sum of the RFs appeared more meaningful than single RFs on their own.

## Supplementary Information

Below is the link to the electronic supplementary material.

Supplementary Material 1

## Data Availability

Data and a codebook (excluding descriptive data) are available at the University of Cambridge repository: https://www.repository.cam.ac.uk/items/e7ac65b9-da6a-44a3-a233-0ade9296fba8.

## References

[1] Kalisch, R., Müller, M. B. & Tüscher, O. A conceptual framework for the neurobiological study of resilience. *Behav. Brain Sci.* **38**, e92 (2015). 25158686 10.1017/S0140525X1400082X

[2] Kalisch, R. et al. The resilience framework as a strategy to combat stress-related disorders. *Nat. Hum. Behav.* **1**, 784–790 (2017). 31024125 10.1038/s41562-017-0200-8

[3] Masten, A. S., Lucke, C. M., Nelson, K. M. & Stallworthy, I. C. Resilience in development and psychopathology: Multisystem perspectives. *Annu. Rev. Clin. Psychol.* **17**, 521–549 (2021). 33534615 10.1146/annurev-clinpsy-081219-120307

[4] Rutter, M. Annual research review: Resilience – clinical implications. *J. Child Psychol. Psychiatry* **54**, 474–487 (2013). 23017036 10.1111/j.1469-7610.2012.02615.x

[5] Troy, A. S. et al. Psychological resilience: An affect-regulation framework. *Annu. Rev. Psychol.* **74**, 547–576 (2023). 36103999 10.1146/annurev-psych-020122-041854 PMC12009612

[6] Fritz, J., de Graaff, A. M. & Caisley, H. A systematic review of amenable resilience factors that moderate and/or mediate the relationship between childhood adversity and mental health in young people. *Front. Psychiatry* **9** (2018). 10.3389/fpsyt.2018.00230 PMC6018532 29971021

[7] Ungar, M. & Theron, L. Resilience and mental health: How multisystemic processes contribute to positive outcomes. *Lancet Psychiatry* **7**, 441–448 (2020). 31806473 10.1016/S2215-0366(19)30434-1

[8] Kalisch, R. et al. Deconstructing and reconstructing resilience: A dynamic network approach. *Perspect. Psychol. Sci.* **14**, 765–777 (2019). 31365841 10.1177/1745691619855637

[9] Fritz, J., Fried, E. I., Goodyer, I. M., Wilkinson, P. O. & Van Harmelen, A.-L. A network model of resilience factors for adolescents with and without exposure to childhood adversity. *Sci. Rep.* **8**, 15774 (2018). 30361515 10.1038/s41598-018-34130-2 PMC6202387

[10] Jefferies, P., Fritz, J., Deighton, J. & Ungar, M. Analysis of protective factors in schoolchildren in England using the dual-factor model of mental health. *Res. Child Adolesc. Psychopathol.* **51**, 907–920 (2023). 36786892 10.1007/s10802-023-01038-z

[11] Schäfer, S. K. et al. Interrelations of resilience factors and their incremental impact for mental health: insights from network modeling using a prospective study across seven timepoints. *Transl. Psychiatry* **13**, 328 (2023). 37872216 10.1038/s41398-023-02603-2 PMC10593776

[12] Wiedemann, A., Gupta, R., Okey, C., Galante, J. & Jones, P. B. A systematic review of pre-pandemic resilience factors and mental health outcomes in adolescents and young adults during the COVID-19 pandemic. *Dev. Psychopathol.*10.1017/S0954579424001901 (2025). 39773864 10.1017/S0954579424001901

[13] Fritz, J., Stochl, J., Goodyer, I. M., van Harmelen, A.-L. & Wilkinson, P. O. Embracing the positive: An examination of how well resilience factors at age 14 can predict distress at age 17. *Transl. Psychiatry* **10**, 272 (2020). 32759937 10.1038/s41398-020-00944-w PMC7406495

[14] Van Der Hallen, R., Jongerling, J. & Godor, B. P. Coping and resilience in adults: a cross-sectional network analysis. *Anxiety Stress Coping* **33**, 479–496 (2020). 32546008 10.1080/10615806.2020.1772969

[15] Churbaji, D., Ritter, L., Schlechter, P., Kip, A. & Morina, N. When an earthquake hits a war-affected population: Longitudinal growth trajectories of psychopathology in individuals in northwest Syria. *Br. J. Psychiatry*10.1192/bjp.2025.1 (2025). 40254687 10.1192/bjp.2025.1 PMC12912877

[16] Ahrens, K. F. et al. Resilience to major life events: Advancing trajectory modeling and resilience factor identification by controlling for background stressor exposure. *Am. Psychol.* **79**, 1076–1091 (2024). 39531708 10.1037/amp0001315

[17] Bonanno, G. A., Westphal, M. & Mancini, A. D. Resilience to loss and potential trauma. *Annu. Rev. Clin. Psychol.* **7**, 511–535 (2011). 21091190 10.1146/annurev-clinpsy-032210-104526

[18] Southwick, S. M., Bonanno, G. A., Masten, A. S., Panter-Brick, C. & Yehuda, R. Resilience definitions, theory, and challenges: Interdisciplinary perspectives. *Eur. J. Psychotraumatology* **5**, 25338 (2014). 10.3402/ejpt.v5.25338 PMC4185134 25317257

[19] Fritz, J., Stochl, J., Kievit, R. A., van Harmelen, A.-L. & Wilkinson, P. O. Tracking stress, mental health, and resilience factors in medical students before, during, and after a stress-inducing exam period: Protocol and proof-of-principle analyses for the resist cohort study. *JMIR Form. Res.* **5**, e20128 (2021). 34100761 10.2196/20128 PMC8262546

[20] Rotenstein, L. S. et al. Prevalence of depression, depressive symptoms, and suicidal ideation among medical students: A systematic review and meta-analysis. *JAMA* **316**, 2214–2236 (2016). 27923088 10.1001/jama.2016.17324 PMC5613659

[21] Dyrbye, L. N., Thomas, M. R. & Shanafelt, T. D. Systematic review of depression, anxiety, and other indicators of psychological distress among U.S. and Canadian medical students. *Acad. Med.* **81**, 354 (2006). 16565188 10.1097/00001888-200604000-00009

[22] Medisauskaite, A., Silkens, M., Lagisetty, N. & Rich, A. UK medical students’ mental health and their intention to drop out: A longitudinal study. *BMJ Open* **15**, e094058 (2025). 39909524 10.1136/bmjopen-2024-094058 PMC11800202

[23] Deakin, M. NHS workforce shortages and staff burnout are taking a toll. *BMJ* **377**, o945 (2022). 35410885 10.1136/bmj.o945

[24] Wunsch, K., Kasten, N. & Fuchs, R. The effect of physical activity on sleep quality, well-being, and affect in academic stress periods. *Nat. Sci. Sleep*10.2147/NSS.S132078 (2017). 28490911 10.2147/NSS.S132078 PMC5414656

[25] Pascoe, M. C., Hetrick, S. E. & Parker, A. G. The impact of stress on students in secondary school and higher education. *Int. J. Adolesc. Youth* **25**, 104–112 (2020).

[26] Stallman, H. M. & Hurst, C. P. The university stress scale: Measuring domains and extent of stress in university students. *Aust. Psychol.* **51**, 128–134 (2016).

[27] Thompson, G., McBride, R. B., Hosford, C. C. & Halaas, G. Resilience among medical students: The role of coping style and social support. *Teach. Learn. Med.* **28**, 174–182 (2016). 27064719 10.1080/10401334.2016.1146611

[28] Rahimi, B., Baetz, M., Bowen, R. & Balbuena, L. Resilience, stress, and coping among Canadian medical students. *Can. Med. Educ. J.* **5**, e5–e12 (2014). 26451221 PMC4563614

[29] Bacchi, S. & Licinio, J. Resilience and psychological distress in psychology and medical students. *Acad. Psychiatry* **41**, 185–188 (2017). 27060093 10.1007/s40596-016-0488-0

[30] Burgis-Kasthala, S., Elmitt, N., Smyth, L. & Moore, M. Predicting future performance in medical students. A longitudinal study examining the effects of resilience on low and higher performing students. *Med. Teach.* **41**, 1184–1191 (2019). 31314633 10.1080/0142159X.2019.1626978

[31] Houpy, J. C., Lee, W. W., Woodruff, J. N. & Pincavage, A. T. Medical student resilience and stressful clinical events during clinical training. *Med. Educ. Online* **22**, 1320187 (2017). 28460570 10.1080/10872981.2017.1320187 PMC5419301

[32] Eley, D. S., Leung, J., Hong, B. A., Cloninger, K. M. & Cloninger, C. R. Identifying the dominant personality profiles in medical students: Implications for their well-being and resilience. *PLoS ONE* **11**, e0160028 (2016). 27494401 10.1371/journal.pone.0160028 PMC4975484

[33] Kjeldstadli, K. et al. Life satisfaction and resilience in medical school—a six-year longitudinal, nationwide and comparative study. *BMC Med. Educ.* **6**, 48 (2006). 16984638 10.1186/1472-6920-6-48 PMC1592096

[34] Leyro, T. M., Zvolensky, M. J. & Bernstein, A. Distress tolerance and psychopathological symptoms and disorders: A review of the empirical literature among adults. *Psychol. Bull.* **136**, 576–600 (2010). 20565169 10.1037/a0019712 PMC2891552

[35] Watkins, E. R. & Roberts, H. Reflecting on rumination: Consequences, causes, mechanisms and treatment of rumination. *Behav. Res. Ther.* **127**, 103573 (2020). 32087393 10.1016/j.brat.2020.103573

[36] Orth, U., Erol, R. Y. & Luciano, E. C. Development of self-esteem from age 4 to 94 years: A meta-analysis of longitudinal studies. *Psychol. Bull.* **144**, 1045–1080 (2018). 30010349 10.1037/bul0000161

[37] Dryman, M. T. & Heimberg, R. G. Emotion regulation in social anxiety and depression: A systematic review of expressive suppression and cognitive reappraisal. *Clin. Psychol. Rev.* **65**, 17–42 (2018). 30064053 10.1016/j.cpr.2018.07.004

[38] You, S. & Lim, S. A. Development pathways from abusive parenting to delinquency: The mediating role of depression and aggression. *Child Abuse Negl.* **46**, 152–162 (2015). 26038191 10.1016/j.chiabu.2015.05.009

[39] Hoagwood, K. E. et al. Family support in children’s mental health: A review and synthesis. *Clin. Child Fam. Psychol. Rev.* **13**, 1–45 (2010). 20012893 10.1007/s10567-009-0060-5

[40] Goodrum, N. M. et al. Longitudinal relations among adolescent risk behavior, family cohesion, violence exposure, and mental health in a national sample. *J. Abnorm. Child Psychol.* **48**, 1455–1469 (2020). 32845455 10.1007/s10802-020-00691-y PMC7530104

[41] Tabak, I. & Zawadzka, D. The importance of positive parenting in predicting adolescent mental health. *J. Fam. Stud.* **23**, 1–18 (2017).

[42] Wang, M. et al. Factor structure of the CES-D and measurement invariance across gender in mainland Chinese adolescents. *J. Clin. Psychol.* **69**, 966–979 (2013). 23775279 10.1002/jclp.21978

[43] Shalaby, R. A. H. & Agyapong, V. I. Peer support in mental health: Literature review. *JMIR Ment. Health* **7**, e15572 (2020). 32357127 10.2196/15572 PMC7312261

[44] Harris, P. A. et al. Research electronic data capture (REDCap)—A metadata-driven methodology and workflow process for providing translational research informatics support. *J. Biomed. Inform.* **42**, 377–381 (2009). 18929686 10.1016/j.jbi.2008.08.010 PMC2700030

[45] Hankins, M. The factor structure of the twelve item General Health Questionnaire (GHQ-12): The result of negative phrasing?. *Clin. Pract. Epidemiol. Ment. Health* **4**, 1–8 (2008). 18435842 10.1186/1745-0179-4-10 PMC2373289

[46] Wojujutari, A. K., Idemudia, E. S. & Ugwu, L. E. The evaluation of the General Health Questionnaire (GHQ-12) reliability generalization: A meta-analysis. *PLoS ONE* **19**, e0304182 (2024). 39018280 10.1371/journal.pone.0304182 PMC11253975

[47] Gnambs, T. & Staufenbiel, T. The structure of the General Health Questionnaire (GHQ-12): Two meta-analytic factor analyses. *Health Psychol. Rev.* **12**, 179–194 (2018). 29325498 10.1080/17437199.2018.1426484

[48] Simons, J. S. & Gaher, R. M. The distress tolerance scale: Development and validation of a self-report measure. *Motiv. Emot.* **29**, 83–102 (2005).

[49] Treynor, W. Rumination reconsidered: A psychometric analysis. *Cogn. Ther. Res.* **27**, 247–259 (2003).

[50] Roelofs, J., Muris, P., Huibers, M., Peeters, F. & Arntz, A. On the measurement of rumination: A psychometric evaluation of the ruminative response scale and the rumination on sadness scale in undergraduates. *J. Behav. Ther. Exp. Psychiatry* **37**, 299–313 (2006). 16678121 10.1016/j.jbtep.2006.03.002

[51] Rosenberg, M. Rosenberg self-esteem scale. *J. Relig. Health* (1965).

[52] Huang, C. & Dong, N. Factor structures of the Rosenberg self-esteem scale. *Eur. J. Psychol. Assess.* **28**, 132–138 (2012).

[53] Gross, J. J. & John, O. P. Individual differences in two emotion regulation processes: Implications for affect, relationships, and well-being. *J. Pers. Soc. Psychol.* **85**, 348–362 (2003). 12916575 10.1037/0022-3514.85.2.348

[54] Preece, D. A., Becerra, R., Robinson, K. & Gross, J. J. The emotion regulation questionnaire: psychometric properties in general community samples. *J. Pers. Assess.* **102**, 348–356 (2020). 30714818 10.1080/00223891.2018.1564319

[55] Webster, G. D. et al. The brief aggression questionnaire: psychometric and behavioral evidence for an efficient measure of trait aggression. *Aggress. Behav.* **40**, 120–139 (2014). 24115185 10.1002/ab.21507

[56] Webster, G. D. et al. The brief aggression questionnaire: Psychometric and behavioral evidence for an efficient measure of trait aggression. *Aggress. Behav.* **40**, 120–139 (2014). 24115185 10.1002/ab.21507

[57] Procidano, M. E. & Heller, K. Measures of perceived social support from friends and from family: Three validation studies. *Am. J. Community Psychol.* **11**, 1–24 (1983). 6837532 10.1007/BF00898416

[58] Lyons, J. S., Perrotta, P. & Hancher-Kvam, S. Perceived social support from family and friends: Measurement across disparate samples. *J. Pers. Assess.* **52**, 42–47 (1988). 3361411 10.1207/s15327752jpa5201_3

[59] Taylor, R. D., Casten, R. & Flickinger, S. M. Influence of kinship social support on the parenting experiences and psychosocial adjustment of African-American adolescents. *Dev. Psychol.* **29**, 382 (1993).

[60] Beavers, W. R. & Hampson, R. B. *Successful Families: Assessment and Intervention* (WW Norton & Co, 1990).

[61] Shelton, K. K., Frick, P. J. & Wootton, J. Assessment of parenting practices in families of elementary school-age children. *J. Clin. Child Psychol.* **25**, 317–329 (1996).

[62] Shelton, K. K., Frick, P. J. & Wootton, J. Assessment of parenting practices in families of elementary school-age children. *J. Clin. Child Psychol.* **25**, 317–329 (1996).

[63] Essau, C. A., Sasagawa, S. & Frick, P. J. Psychometric properties of the Alabama parenting questionnaire. *J. Child Fam. Stud.* **15**, 595–614 (2006).

[64] Graham, J. W. Missing data analysis: Making it work in the real world. *Annu. Rev. Psychol.* **60**, 549–576 (2009). 18652544 10.1146/annurev.psych.58.110405.085530

[65] R Core Team. *R: A Language and Environment for Statistical Computing. *(R Foundation for Statistical Computing, Vienna: Austria 2025).

[66] Tonidandel, S. & LeBreton, J. M. Relative importance analysis: A useful supplement to regression analysis. *J. Bus. Psychol.* **26**, 1–9 (2011).

[67] Lindeman, R. H., Merenda, P. F. & Gold, R. Z. *Introduction to Bivariate and Multivariate Analysis*. https://cir.nii.ac.jp/crid/1130282269700928896 (1980).

[68] Groemping, U. & Matthias, L. Package ‘relaimpo’. *Relat. Importance Regressors Linear Models R Package Version* http://r.meteo.uni.wroc.pl/web/packages/relaimpo/relaimpo.pdf (2018).

[69] Cohen, J. *Statistical Power Analysis for the Behavioral Sciences* (Erlbaum, 1988).

[70] Ehring, T. Thinking too much: Rumination and psychopathology. *World Psychiatry* **20**, 441–442 (2021). 34505392 10.1002/wps.20910 PMC8429319

[71] Holliday, S. B., Pedersen, E. R. & Leventhal, A. M. Depression, posttraumatic stress, and alcohol misuse in young adult veterans: The transdiagnostic role of distress tolerance. *Drug Alcohol Depend.* **161**, 348–355 (2016). 26948757 10.1016/j.drugalcdep.2016.02.030 PMC4792662

[72] McLaughlin, K. A. & Nolen-Hoeksema, S. Rumination as a transdiagnostic factor in depression and anxiety. *Behav. Res. Ther.* **49**, 186–193 (2011). 21238951 10.1016/j.brat.2010.12.006 PMC3042543

[73] Orth, U. & Robins, R. W. The development of self-esteem. *Curr. Dir. Psychol. Sci.* **23**, 381–387 (2014).

[74] Sowislo, J. F. & Orth, U. Does low self-esteem predict depression and anxiety? A meta-analysis of longitudinal studies. *Psychol. Bull.* **139**, 213–240 (2013). 22730921 10.1037/a0028931

[75] Carey, E. G., Ridler, I., Ford, T. J. & Stringaris, A. Editorial perspective: When is a ‘small effect’actually large and impactful?. *J. Child Psychol. Psychiatry*10.1111/jcpp.13817 (2023). 37226639 10.1111/jcpp.13817

[76] Ding, F. & Yu, B. First year university students’ perception of autonomy: An individualistic approach. *J. Furth. High. Educ.* **46**, 211–224 (2022).

[77] Bonanno, G. A. & Westphal, M. The three axioms of resilience. *J. Trauma. Stress* **37**, 717–723 (2024). 38840482 10.1002/jts.23071

[78] Fritz, J. et al. So you want to do ESM? 10 essential topics for implementing the experience-sampling method. *Adv. Methods Pract. Psychol. Sci.* **7**, 25152459241267910 (2024).

[79] Fritz, J. et al. So you want to do ESM? Ten Essential Topics for Implementing the Experience Sampling Method (ESM). https://osf.io/fverx/download (2024).

[80] Schlechter, P., Meyer, T., Hagen, M., Baranova, K. & Morina, N. Comparative thinking among university students: An ecological momentary assessment of upward comparisons, stress and learning behavior during exam preparation. *Soc. Psychol. Educ.* **28**, 54 (2025).

