# Supplementary material for: The relative importance of resilience factors prior, during, and after stress-inducing exams in medical students
Source: Sci Rep. 2026 Jul 24;16:23221. doi: 10.1038/s41598-026-63690-x (PMC13400740; doi:10.1038/s41598-026-63690-x)
Supplement: Supplementary file 1 — Supplementary Material 1 [file 41598_2026_63690_MOESM1_ESM.docx]

**Supplemental Materials for:**

**The Relative Importance of Resilience Factors**

**Prior, During, and After Stress-Inducing Exams in Medical Students**

Pascal Schlechter^1^, Paul O. Wilkinson^2^, Philip Jefferies^3^, & Jessica Fritz^4^

^1^Institute of Psychology, University of Münster, Germany

^2^Department of Psychiatry, University of Cambridge, UK

^3^School of Psychology, Atlantic Technological University, Ireland

^4^Institute of Psychology, University of Osnabrück, Germany

**Short Title:** Resilience Factors Prior, During, and After Stress-Inducing Exams Correspondence concerning this article should be addressed to Pascal Schlechter, Institute of Psychology, University of Münster, Germany, E-mail: p_schl20@uni-muenster.de.

**Declarations**

**Competing Interests** The authors declare no competing interests

**Funding Statement** RESIST was approved by the Cambridge Psychology Research Ethics Committee (PRE.2017.096). RESIST was funded by J. Fritz’s Medical Research Council Doctoral Training Grant and by P. O. Wilkinson’s personal research account.

**Ethical Approval Statement:** Participants provided informed consent and the study was approved by the University of Cambridge Research Ethics Committee (PRE.2017.096).

**Data Availability Statement:** Data and a codebook (excluding descriptive data) are available at the University of Cambridge repository: https://www.repository.cam.ac.uk/items/e7ac65b9-da6a-44a3-a233-0ade9296fba8.

| Model |  | *RI A Models* |  |  | *RI B Models* |  |  | *RI C Models* |  |
| --- | --- | --- | --- | --- | --- | --- | --- | --- | --- |
| Timepoints  Explained total variance | T1 -> T2  R² = .28 | T1 -> T3  R² = .13 | T2 -> T3  R² = .09 | T1 -> T2  R² = .36 | T1 -> T3  R² = .16 | T2 -> T3  R² = .10 | T1 -> T2  R² = .28 | T1 -> T3  R² = .12 | T2 -> T3  R² = .08 |
| Sum of all resilience factors | - | - | - | .22 | .10 | .06 | .04 | .05 | .04 |
| Baseline mental health | - | - | - | .14 [.05; .23] | .04 [.00; .13] | .02 [.00; .13] | .22[.15; .29] | .05 [.01; .09] | .04 [.01; .10] |
| High distress tolerance | .06 [.02; .14] | .02 [.00; .10] | .02 [.00; .10] | .05 [.01; .11] | .01 [.00; .08] | .02 [.00; .09] | .01 [.00; .04] | .00 [.00; .06] | .01 [.00; .07] |
| Low ruminative reflection | .01 [.00; .05] | .01 [.00; .09] | .00 [.00; .05] | .01 [.00; .04] | .01 [.00; .07] | .00 [.00; .06] | .00 [.00; .03] | .01 [.00; .08] | .00 [.00; .07] |
| Low ruminative brooding | .06 [.01; .14] | .01 [.00; .05] | .01 [.00; .07] | .04 [.01; .13] | .01 [.00; .04] | .01 [.00; .04] | .01 [.00; .04] | .00 [.00; .04] | .01 [.00; .05] |
| High self-esteem | .09 [.03; .18] | .03 [.00; .15] | .02 [.00; .11] | .07 [.03; .15] | .02 [.00; .12] | .01 [.00; .09] | .01 [.00; .07] | .00 [.00; .07] | .01 [.00; .06] |
| High cognitive reappraisal | .01 [.00; .06] | .00 [.00; .04] | .00 [.00; .07] | .01 [.00; .05] | .00 [.00; .04] | .00 [.00; .06] | .00 [.00; .03] | .00 [.00; .06] | .00 [.00; .06] |
| Low expressive suppression | .00 [.00; .05] | .00 [.00; .06] | .02 [.00; .09] | .00 [.00; .04] | .00 [.00; .04] | .01 [.00; .09] | .00 [.00; .04] | .00 [.00; .05] | .01 [.00; .07] |
| Low aggression potential | .00 [.00; .04] | .00 [.00; .05] | .00 [.00; .07] | .00 [.00; .05] | .00 [.00; .05] | .00 [.00; .07] | .00 [.00; .04] | .00 [.00; .05] | .00 [.00; .06] |
| High immediate family support | .01 [.00; .08] | .04 [.00; .11] | .00 [.00; .06] | .01 [.00; .06] | .03 [.00; .12] | .00 [.00; .06] | .00 [.00; .06] | .02 [.00; .08] | .00 [.00; .06] |
| High extended family support | .00 [.00; .03] | .00 [.00; .04] | .00 [.00; .04] | .00 [.00; .03] | .00 [.00; .04] | .00 [.00; .04] | .00 [.00; .03] | .00 [.00; .04] | .00 [.00; .05] |
| High family cohesion | .00 [.00; .04] | .01 [.00; .07] | .00 [.00; .09] | .00 [.00; .03] | .01 [.00; .06] | .00 [.00; .09] | .00 [.00; .03] | .01 [.00; .07] | .00 [.00; .08] |
| High positive parenting | .02 [.00; .06] | .01 [.00; .07] | .00 [.00; .05] | .02 [.00; .06] | .01 [.00; .05] | .00 [.00; .06] | .01 [.00; .05] | .01 [.00; .05] | .00 [.00; .05] |
| High parental involvement | .01 [.00; .07] | .00 [.00; .06] | .00 [.00; .06] | .01 [.00; .07] | .00 [.00; .05] | .00 [.00; .07] | .00 [.00; .06] | .00 [.00; .05] | .00 [.00; .07] |
| High friendship support | .00 [.00; .04] | .00 [.00; .06] | .01 [.00; .04] | .00 [.00; .03] | .00 [.00; .04] | .01 [.00; .04] | .00 [.00; .04] | .00 [.00; .03] | .00 [.00; .05] |

**Table s1**

*Relative Importance of the resilience factors on mental health outcome based on full cases*

*Note.* Number in brackets refer to bootstrapped 99.9% confidence intervals based in 1,000 iterations. Relative Importance of the resilience factors without autoregressive factors in regression models (RI A Models); Relative Importance of the resilience factors and autoregressive effect of baseline mental health in regression models (RI B Models); Relative Importance of the resilience factors with autoregressive effect of baseline mental health always accounted for (RI C Models).

*Effect size rounded to R² < 0.015 are considered as negligible, 0.015 ≤ R² < 0.09 as small, 0.09 ≤ R² < 0.25 as moderate, and R² > 0.25 as large.*

**Supplemental Figure s1**

*Correlation matrix of resilience factors at timepoint 1*


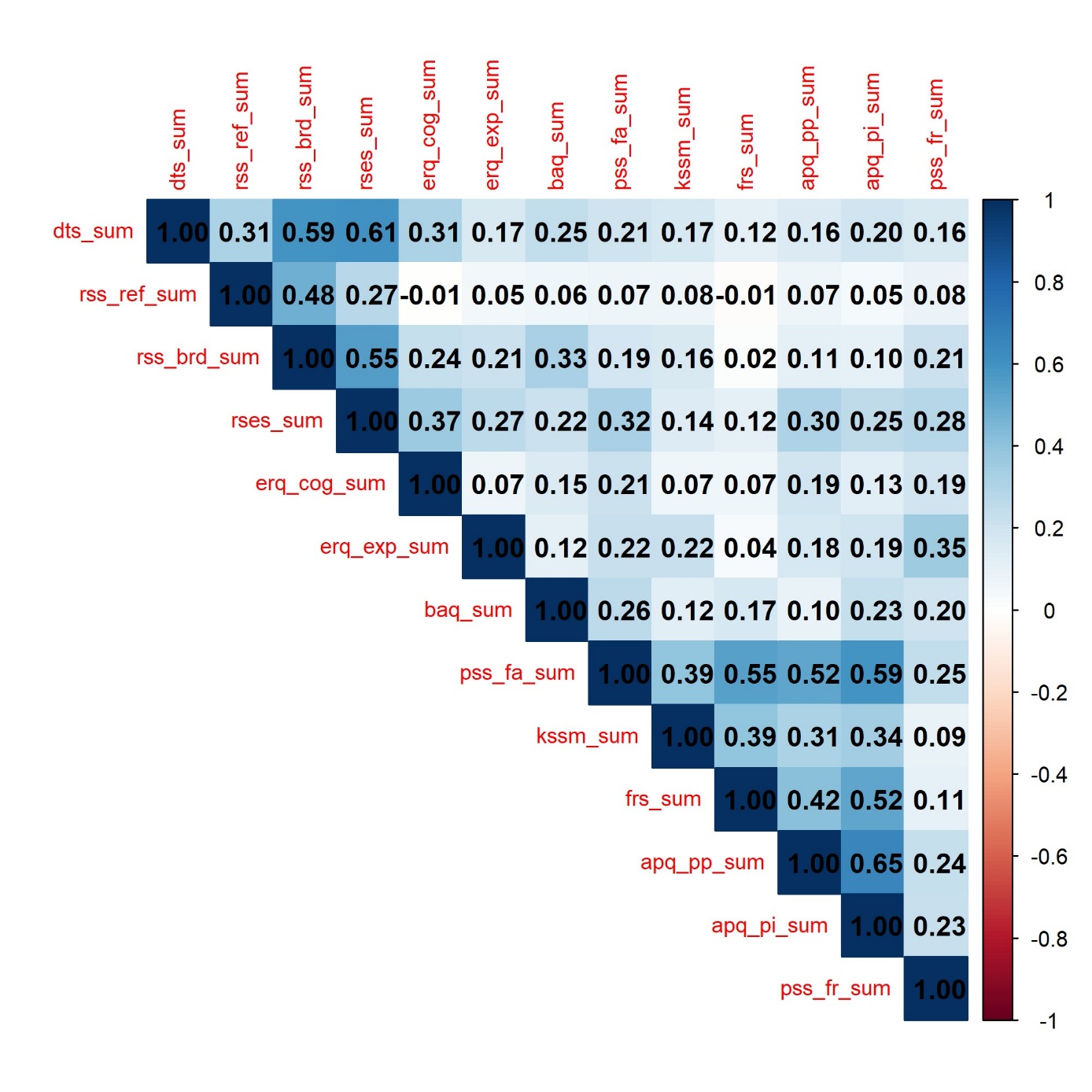


Note. DTS = Distress Tolerance Scale (subjective appraisal of distress subscale); RRS = Ruminative Response Scale (with RRS-Ref = reflective rumination and RRS-Bro = brooding subscales); RSES = Rosenberg Self-Esteem Scale; ERQ-Cog = Emotion Regulation Questionnaire cognitive reappraisal subscale; ERQ-Exp = Emotion Regulation Questionnaire expressive suppression subscale; BAQ = Brief Aggression Questionnaire; PSS-FA = Perceived Social Support from Family Scale (immediate family support); KSSM = Kinship Social Support Measure (extended family support); FRS = family cohesion subscale of the Self-Report Family Inventory; APQ-PP = Alabama Parenting Questionnaire positive parenting subscale; APQ-PI = Alabama Parenting Questionnaire parental involvement subscale; PSS-FR = Perceived Social Support from Friends Scale (friendship support).

**Supplemental Figure s2**

*Correlation matrix of resilience factors at timepoint 2*


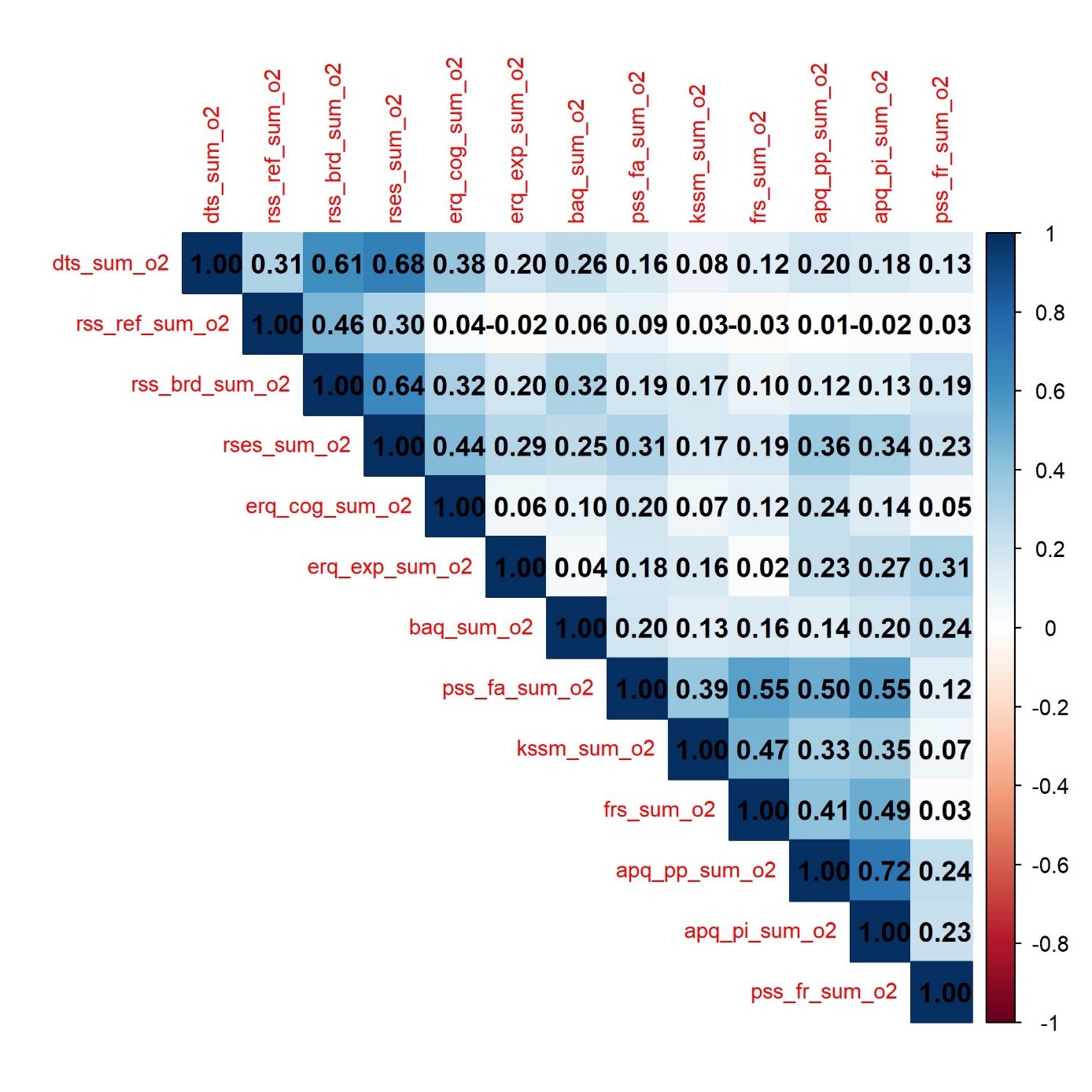


Note. DTS = Distress Tolerance Scale (subjective appraisal of distress subscale); RRS = Ruminative Response Scale (with RRS-Ref = reflective rumination and RRS-Bro = brooding subscales); RSES = Rosenberg Self-Esteem Scale; ERQ-Cog = Emotion Regulation Questionnaire cognitive reappraisal subscale; ERQ-Exp = Emotion Regulation Questionnaire expressive suppression subscale; BAQ = Brief Aggression Questionnaire; PSS-FA = Perceived Social Support from Family Scale (immediate family support); KSSM = Kinship Social Support Measure (extended family support); FRS = family cohesion subscale of the Self-Report Family Inventory; APQ-PP = Alabama Parenting Questionnaire positive parenting subscale; APQ-PI = Alabama Parenting Questionnaire parental involvement subscale; PSS-FR = Perceived Social Support from Friends Scale (friendship support).

**Supplemental Figure s3**.

*Correlation matrix of resilience factors at timepoint 3*


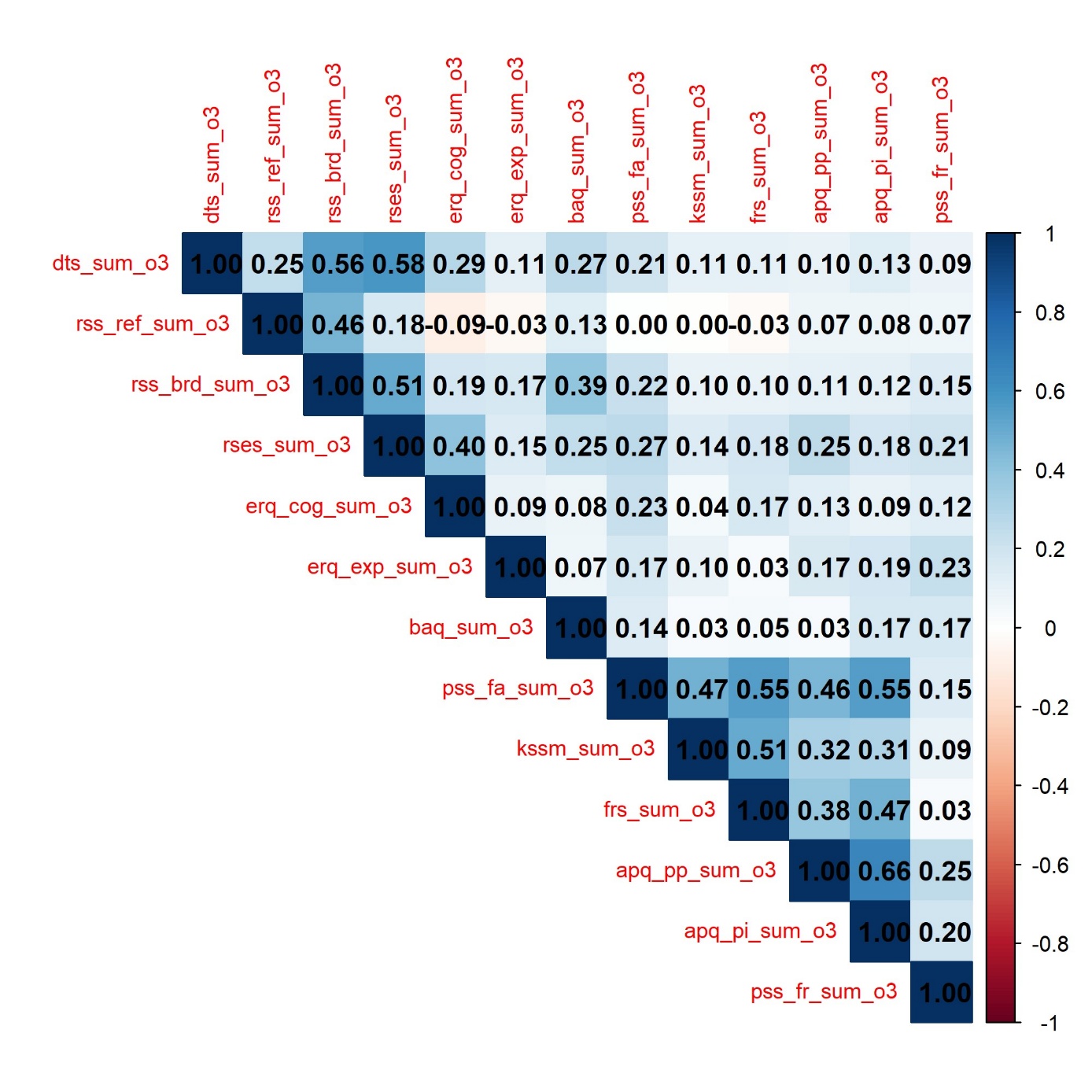


Note. DTS = Distress Tolerance Scale (subjective appraisal of distress subscale); RRS = Ruminative Response Scale (with RRS-Ref = reflective rumination and RRS-Bro = brooding subscales); RSES = Rosenberg Self-Esteem Scale; ERQ-Cog = Emotion Regulation Questionnaire cognitive reappraisal subscale; ERQ-Exp = Emotion Regulation Questionnaire expressive suppression subscale; BAQ = Brief Aggression Questionnaire; PSS-FA = Perceived Social Support from Family Scale (immediate family support); KSSM = Kinship Social Support Measure (extended family support); FRS = family cohesion subscale of the Self-Report Family Inventory; APQ-PP = Alabama Parenting Questionnaire positive parenting subscale; APQ-PI = Alabama Parenting Questionnaire parental involvement subscale; PSS-FR = Perceived Social Support from Friends Scale (friendship support).
